# Mis-Spliced *Lr34* Transcript Events in Winter Wheat

**DOI:** 10.1371/journal.pone.0171149

**Published:** 2017-01-30

**Authors:** Tilin Fang, Brett F. Carver, Robert M. Hunger, Liuling Yan

**Affiliations:** 1 Department of Plant and Soil Sciences, Oklahoma State University, Stillwater, Oklahoma, United States of America; 2 Department of Entomology and Plant Pathology, Oklahoma State University, Stillwater, Oklahoma, United States of America; Institute of Genetics and Developmental Biology Chinese Academy of Sciences, CHINA

## Abstract

*Lr34* in wheat is a non-race-specific gene that confers resistance against multiple fungal pathogens. The resistant allele *Lr34* and the susceptible allele *Lr34s* can be distinguished by three polymorphisms that cause alternation of deduced amino acid sequences of Lr34 at the protein level. In seedlings of a cultivar carrying the resistant *Lr34r* allele, only a portion (35%) of its transcripts was correctly spliced and the majority (65%) of its transcripts were incorrectly spliced due to multiple mis-splicing events. *Lr34* mis-splicing events were also observed at adult plant age when this gene exerts its function. All of the mis-spliced *Lr34r* cDNA transcripts observed in this study resulted in a premature stop codon due to a shift of the open reading frame; hence, the mis-spliced *Lr34r* cDNAs were deduced to encode incomplete proteins. Even if a cultivar has a functional *Lr34* gene, its transcripts might not completely splice in a correct pattern. These findings suggested that the partial resistance conferred by a quantitative gene might be due to mis-splicing events in its transcripts; hence, the resistance of the gene could be increased by eliminating or mutating regulators that cause mis-splicing events in wheat.

## Introduction

Wheat (*Triticum aestivum*, 2n = 6x = 42, AABBDD) is a major food crop worldwide, but is constantly challenged by several fungal diseases, such as leaf rust and stripe rust [1, 2]. There are numerous genes or quantitative trait loci (QTL) that have been mapped for host plant resistance to epidemic pathogens, including 67 genes/QTL for stripe rust and 69 for leaf rust [3]. The resistance of these genes/QTL can be divided into two types: complete or qualitative resistance, and partial or quantitative resistance [4–11]. Qualitative resistance fits a gene-for-gene model, in which a pathogen is recognized by a plant host resistance gene resulting in complete exclusion of the pathogen [12]. Quantitative resistance remains poorly understood but is explained by two hypotheses: i) a consequence of interactions among multiple resistance genes [13, 14], or ii) the outcome of direct interactions between pathogen effectors and plant defense proteins or indirect interactions between pathogen effectors and plant defense proteins triggered by the effectors [15, 16].

*Lr34* is a non-race-specific gene that confers resistance against fungal pathogens causing leaf rust, stripe rust, and powdery mildew, but also against barley yellow dwarf virus [17–20]. Because of the durability and non-specificity of its resistance against multiple pathogens, *Lr34* has become one of the most important disease-resistance genes in wheat worldwide and has been utilized since the early 20^th^ century [21–23]. Cloning of *Lr34* indicated that the resistant allele of the gene consists of 24 exons and 23 introns spanning 11,805 nucleotide sequence from the translational start codon to the stop codon, and it encodes a pleiotropic drug resistance (PDR)-like adenosine triphosphate-binding cassette (ABC) transporter, which consists of two units each containing a cytosolic nucleotide binding domain (NBD) and a hydrophobic transmembrane domain (TD) [2]. The resistant allele *Lr34r* and the susceptible allele *Lr34s* can be distinguished by three polymorphisms. The first is the codon TTC encoding phenylalanine is present in exon 11 of the susceptible *Lr34E11s* allele but absent in the resistant *Lr34E11r* allele, and the second is that a point mutation in exon 12 from ‘C’ to ‘T’ results in a change from a tyrosine residue in the susceptible Lr34E12s protein to a histidine residue in the resistant Lr34E12r protein [2, 19]. The third is that susceptible *Lr34E22s* contains a point mutation in exon 22 that results in a premature stop codon and thus a shortened Lr34E22s protein lacking 185 amino acids covering the majority of the second transmembrane domain [2, 19, 22, 24]. All three mutations resulted in alternation of deduced amino acid sequences of Lr34r at the protein level. Understanding of *Lr34r* transcriptional defects will contribute significantly to establish a new strategy to improve *Lr34* resistance.

Our previous studies on winter wheat cultivars adapted to the southern Great Plains indicated that *Lr34* accounted for 18 to35% of the total phenotypic variation in leaf rust reaction among recombinant inbred lines (RILs) from the biparental cross of ‘Jagger’ carrying a susceptible *Lr34E22s* allele and ‘2174’ carrying a resistant *Lr34E22r* allele [22]. *Lr34* was also reported to account for 29 to 33% of the variance for leaf rust reaction in other studies [2, 14, 25]. However, the molecular mechanism for the partial resistance conferred by this gene remains largely unknown.

The deployment of the *Lr34* gene in wheat breeding is still based on the conventional breeding approach, by which a resistant allele from a donor is introduced to a recipient cultivar carrying a susceptible allele. In this study we report that the partial resistance of an endogenous *Lr34* occurs because the majority of its transcripts are incorrectly spliced due to intron retention or exon skipping. This finding leads to a novel strategy of breeding cultivars resistant to rust pathogens, in which regulators that result in mis-spliced transcripts of *Lr34* are mutated to increase endogenous *Lr34* transcript level and thus resistance to multiple diseases in wheat.

## Materials and Methods

### Plant materials

The leaf samples, which were found in the initial experiment to have mis-spliced *Lr34* transcripts, were collected from 2174 seedlings that were grown in a greenhouse with constant temperature (20–25°C) and long day condition (16 hour/8 hour for light/dark). Those fully developed leaves at plants the 4^th^ or 5^th^ leaf stage were sampled.

Nine RILs carrying the resistant *Lr34* allele from the Jagger × 2174 RIL population were selected to examine mis-spliced *Lr34* transcripts, and these nine RILs were selected based on their percent infection score averaged across three environments/years [22]. These nine RILs were vernalized for 6 weeks at 4°C and long days then moved to the same greenhouse as conditioned with constant temperature and long day. The RNA samples were collected one week after the vernalized RILs were moved back to the greenhouse and the plants were at the 4^th^ or 5^th^ leaf stage. The RNA samples were collected from the same set of the nine RILs that were continuously grown in the same greenhouse until the 4^th^ or 5^th^ leaf stage as control (CK). Those fully developed leaves at vernalized and unvernalized seedling plants were sampled for RNAs.

Adults of four wheat cultivars, including 2174, Jagger, OK bullet, and Duster that were grown in a field trial at Stillwater Research Station, Oklahoma, were investigated for mis-spliced *Lr34* transcripts. Leaf samples from the adult plants were collected from flag leaves when the plants were flowering (May 5, 2011). All of the adult plants grown in the field were not inoculated with any pathogens.

### Sequences of clones for mis-spliced *Lr34* transcripts

Total RNAs were isolated using TRIZOL and RNAs were reverse transcribed by SuperScript II reverse Transcriptase (Invitrogen, Grand Island, NY, USA). The paired primers Lr34-Exp-F1 (5’-TAGCAAAGGGCGTCGATTTA-3’) and Lr34-Exp-R1 (5’- CTGTTGGAATGTCAGAACTTGC-3’) were designed to amplify complete cDNA of *Lr34* including 36 bp at the 5’ UTR and 22 bp at the 3’ UTR. PCR was performed using LongAmp Taq DNA polymerase (New Englan Biolabs, Ipswich, MA, USA). PCRs were performed using a cycling program consisting of a denaturation cycle at 94°C for 5 min; 40 cycles of amplification (94°C for 30 sec, 60°C for 4 min, 72°C for 30 sec per cycle), and a final extension cycle of 72°C for 10 min. The PCR products of ~4.2 kb cDNAs were then run at a 1% low melting gel, purified by Wizard SV Gel and PCR Clean-Up system (Promega, Madison, WI, USA), and cloned into pGEM-T vector (Promega, Madison, WI, USA). Plasmid DNAs from single clones were completely sequenced.

### Two PCR markers for mis-spliced *Lr34* transcripts

After different forms of mis-spliced *Lr34* transcripts were found, two pairs of primers were designed to investigate for two frequently occurred mis-spliced *Lr34* transcripts. Primers Lr34-DMS-F2 (5’-AGATGATTGTGGGCCCCGCAAGT-3’) and Lr34-DMS-R2 (5’-TTACGAGTGCAATAATGGCAAGCTG-3’) were designed to examine the skipping of 92 bp at the 5’ end of exon 10, and the PCR product size from normal *Lr34* transcripts was 597 bp, and partial E10 skipping fragment was 505 bp. Lr34-DMS-F3 (5’-CTATATGGGAGCATTATTTTTTTCCATCATG -3’) and Lr34-DMS-R3 (5’- AACCATCCTGGCATGGAGGTCT-3’) were designed to examine the skipping of 44 bp at the 5’ end of partial exon 12. For partial E12 skipping, the normal *Lr34* transcript was 414 bp, and the fragment with skipped partial exon 12 was 370 bp. Those primers specific to *Lr34* were not able to amplify gDNAs, if any was contaminated in the cDNA samples, because one of each pair covered two exons. PCRs for the mis-spliced *Lr34* markers were performed using a cycling program consisting of a denaturation cycle at 94°C for 5 min; 40 cycles of amplification (94°C for 30 sec, 55°C for 30 sec, 72°C for 30 sec per cycle), and a final extension cycle of 72°C for 10 min.

## Results

### The majority of *Lr34* transcripts in seedlings were mis-spliced

In an initial experiment of this study, we attempted to amplify a complete cDNA of *Lr34r* that can be used to create transgenic wheat. Winter wheat cultivar 2174 that has been experimentally confirmed to have a resistant allele at *Lr34-Dr* on homoeologous chromosome 7D [22] was used to amplify the complete cDNA of *Lr34-Dr*. At the beginning of the study, no sequence for homoeologous *Lr34* genes in common wheat was available, primers Lr34-Exp-F1 and Lr34-Exp-R1 primers used to amplify *Lr34r* on chromosome 7D were found to amplify *Lr34-B* that was translocated to homoeologous genome 4A in hexaploid wheat [26]. A total of 43 clones were sequenced using primer M13 from cloning vector, 23 clones were found to come from *Lr34* and the remaining 20 clones were found to come from *Lr34-B*. The sequence of a complete *Lr34-B* in 2174 (S1 Fig) is putative functional, which is similar to the *Lr34-B* in wheat cultivar ‘Chinese Spring’ that was reported to be translocated to chromosome 4A [26]. Only the sequences from *Lr34* were further analyzed in this study.

**Fig 1:**
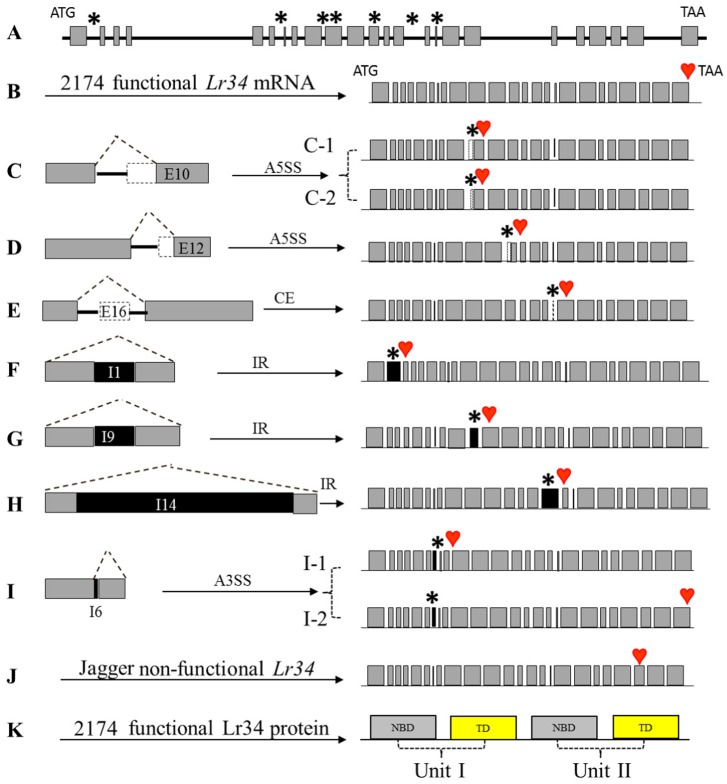
Structure of mis-spliced cDNAs derived from *Lr34*. **(A)**, The *Lr34* gene structure consisting of 24 exons and 23 introns. **(B)**, The correctly spliced *Lr34* mRNA. **(C-I)**, the left panels: locations of alternative splicing events described as skipped or cassette exon (CE), intron retention (IR), alternative 3’ splicing sites (A3SS), and alternative 5’ splicing sites (A5SS). **(C)**, Partial exon 10 skipping, 92 bp (C-1) and 11 bp (C-2) at the 5’ end of exon 10. **(D)**, 44 bp at the 5’ end of exon 12 skipping. **(E)**, Complete exon 16 skipping. **(F)**, Complete intron 1 retention; **(G)**, Complete intron 9 retention. **(H)**, Complete intron 14 retention. **(I)**, Intron 6 was partially retained with 12 bp (I-1) or 17 bp (I-2) at the 3’ end of this intron respectively. **(C-I)**, the right panels, deduced proteins of *Lr34*. Grey boxes indicate exons, lines indicate introns, dotted line boxes indicate the skipped exons, black boxes indicate the retained introns, stars indicate mis-spliced fragments in mRNA, and hearts indicate the premature stop codon. **(J)**, The non-functional Lr34 of Jagger. **(K)**, Protein structure of functional Lr34 of 2174, consisting of two units each containing a cytosolic nucleotide binding domain (NBD) and a hydrophobic transmembrane domain (TD).

A resistant *Lr34r* gene consists of 24 exons and 23 introns in 2174 (Fig 1A), and its cDNA would be functional if all of its introns were spliced out and exons were retained and assembled in a correct pattern (Fig 1B). Surprisingly, however, 15 (65%) cDNAs of the 23 *Lr34r* cDNA clones were found incorrectly spliced (S1 Table). All of these clones were from the same RNA samples collected from complete leaves of the 2174 seedlings grown in the constant temperature and long days, and the plants were at the 4^th^ or 5^th^ leaf stage.

**Fig 2:**
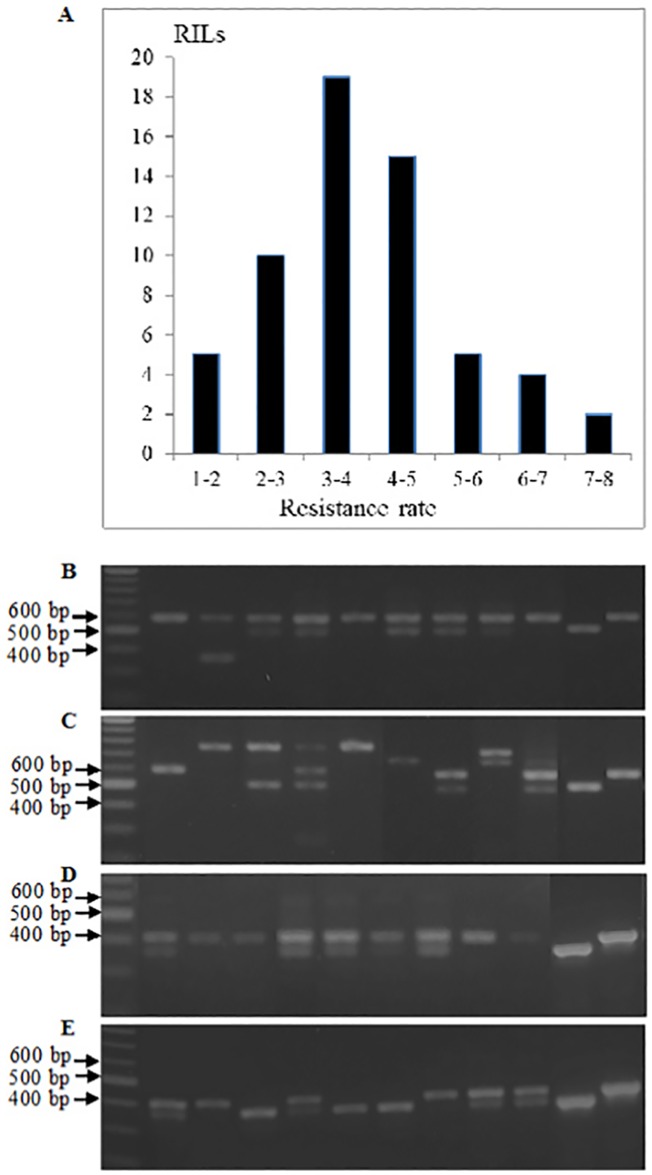
Comparison of patterns of mis-spliced transcripts among RILs. **(A)**, Frequency distribution of nine RILs carrying the resistant *Lr34* allele selected from the Jagger × 2174 RIL population for percent infection score averaged across three environments/years. **(B)**, Expression of normal and mis-spliced *Lr34* using primers Lr34-DMS-F2 and Lr34-DMS-R2 to amplify regions covering exon 10 (E10) and primers Lr34-DMS-F2 and Lr34-DMS-R2 exon 12 (E12). M: DNA ladder marker; Lane 1–9: 9 lines that have the resistant *Lr34* allele in the RILs population. Lane 10: mis-spliced cDNA (PCR products from the plasmid containing mis-spliced *Lr34* cDNA). Lane 11: normal cDNA (PCR products from the plasmid containing normal *Lr34* cDNA. CK indicates plants that were grown in greenhouse as control, and LT indicates plants that were treated with low temperature.

From the 15 mis-spliced *Lr34* clones, nine unique mis-splicing events were observed, including four involving exon skipping and five involving intron retention. The exon skipping events included two partial exon skipping at the same exon: 92 bp (Fig 1C-1) or 11bp (Fig 1C-2) at the 5’ end of exon 10, a partial exon skipping of 44 bp at the 5’ end of exon 12 (Fig 1D), and a complete exon skipping of exon 16 (Fig 1E). The intron retention events included three introns that were completely retained: intron 1 (99 bp) (Fig 1F), intron 9 (92 bp) (Fig 1G), and intron 14 (437 bp) (Fig 1H), and intron 6 that was partially retained with 12 bp or 17 bp at the 3’ end of this intron (Fig 1I, S2 Fig).

The 15 mis-spliced *Lr34r* cDNAs included 11 cDNAs that had a single event of exon skipping or intron retention and 4 cDNAs that had combination of different mis-splicing events (S1 Table). The most frequent mis-splicing events were a skipping of 92 bp at the 5’ end of exon 10 (6 clones) and 44 bp at the 5’ end of exon 12 (5 clones). A multiple sequence alignment of 15 mis-spliced *Lr34* sequences is shown in S3 Fig. Since the sequenced cDNA clones may not include a mis-spliced transcript that was expressed at a low transcriptional level, it is anticipated that many more mis-splicing events exist in *Lr34r* transcripts.

### The mis-splicing of *Lr34r* resulted in loss of interaction site at the protein level

A complete *Lr34r* cDNA encodes a functional Lr34r protein consisting of two units, each containing one cytosolic nucleotide binding domain (NBD) and one hydrophobic transmembrane domain (TD) (Fig 1J) [2, 26]. All of the mis-spliced *Lr34r* cDNA transcripts observed in this study resulted in a premature stop codon due to a shift of the open reading frame; hence, the mis-spliced *Lr34r* cDNAs were deduced to encode incomplete proteins (Fig 1C to 1I-1), but with one exception that was deduced to have an insertion of three amino acids in the full protein due to retention of 12 bp at the 3’ end of intron 6 (Fig 1I-2).

A G/T polymorphism occurs in exon 22, resulting in a non-functional protein due to a premature stop codon and the lack of 185 amino acids at the C terminal in the Lr34s protein encoded by the Jagger *Lr34s* allele (Fig 1J) compared with the 2174 *Lr34r* allele (Fig 1B). The deduced proteins from the mis-spliced cDNAs observed in this study (Fig 1C to 1I-1) were even shorter than the Jagger Lr34s protein (Fig 1J) and did not include the 2^nd^ NBD-TD unit present in the functional protein (Fig 1K); hence, the truncated Lr34 proteins deduced from the mis-spliced cDNAs are unlikely to be functional in resistance to leaf rust.

### Regulation of mis-spliced *Lr34* transcripts by genetic, environmental and developmental factors

*Lr34* transcripts in seedlings were unexpectedly found to be incorrectly spliced, leading us to test if the mis-splicing events occurred in the RNA samples of the plants grown in the specific temperature-photoperiod regime only or these events also occurred in winter wheat plants that were vernalized or in adult plant age when *Lr34r* exerts its function. We designed two pairs of specific primers to detect mis-spliced *Lr34* transcripts that more frequently occurred in the 2174 plants tested in the initial experiment. Primers Lr34-DMS-F2 and Lr34-DMS-R2 were used to examine the skipping of 92 bp at the 5’ end of exon 10, and Lr34-DMS-F3 and Lr34-DMS-R3 were used to examine the skipping of 44 bp at the 5’ end of exon 12.

First, we selected nine RILs from the Jagger x 2174 mapping population (Fig 2A), and all of the nine lines were identified to have the 2174 resistant allele of *Lr34* but representative of the variability in leaf rust reaction that were identified in the field [23]. When the nine RILs were grown in the same temperature-photoperiod controlled regime as tested in the initial experiment (CK), the exon skipping at exon 10 (E10) (Fig 2B) and exon 12 (E12) (Fig 2D) were detectable in agarose gels. The proportion of mis-spliced transcripts was similar among RILs (Fig 2B).

When the RILs were treated with low temperature (LT) for 6 weeks and then moved to the same temperature-photoperiod controlled greenhouse for one week, however, the proportion of the exon 10 skipped transcripts varied from undetectable mis-splicing (lane 2, Fig 2C) to completely mis-spliced transcripts (lane 3, Fig 2C). Furthermore, new patterns of mis-spliced transcripts were observed in the agarose gel. Sequencing results of PCR products from two bands which migrated higher than expected (lane 9, Fig 2C) indicated that one had retained intron 9 and the other had retained both introns 9 and 10. When the plants were treated with low temperature, the proportion of the mis-spliced exon 12 varied widely from undetectable mis-splicing (lanes 2 and 7, Fig 2E) to completely mis-spliced transcripts (lanes 3, 5 and 6, Fig 2E). These resulted indicated that the proportion of mis-spliced exon 10 and exon 12 in the total transcripts was regulated by low temperature.

**Fig 3:**
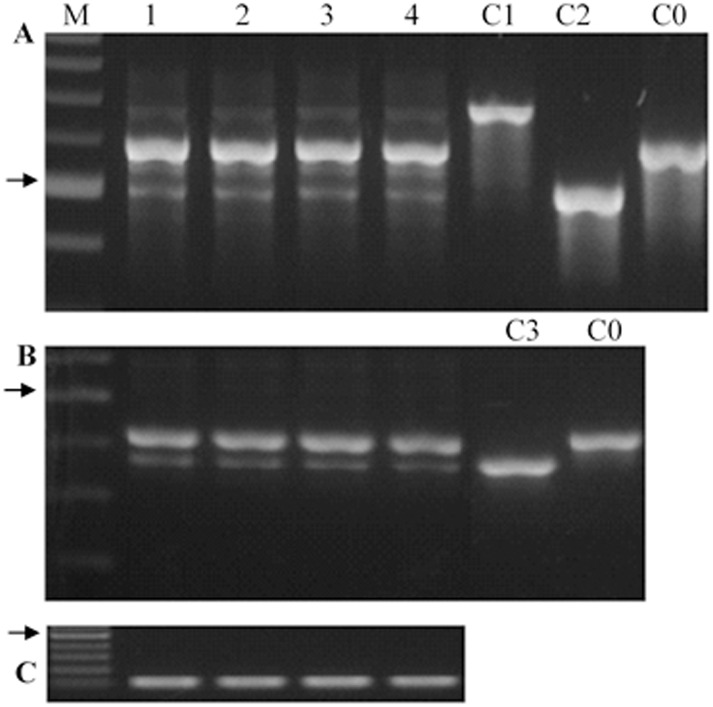
Mis-spliced Lr34 transcripts in wheat cultivars. **(A)**, Mis-spliced Lr34 transcripts using primers Lr34-DMS-F2 and Lr34-DMS-R2 to amplify regions covering exon 10. **(B)**, Mis-spliced Lr34 transcripts using primers Lr34-DMS-F3 and Lr34-DMS-R3 to amplify regions covering exon 12. (**C**), actin expression as control. M. Marker (arrow showing 500-bp fragment); 1. Jagger; 2. 2174; 3. OK Bullet; 4. Duster; C1: clone 1 that has intron 9 retention; C2: clone 2 that has partial exon 10 (92 bp) skipped; C3: clone 3 that has partial exon 12 (44 bp) skipped; C0: clone that has correct spliced exons and introns.

Nest, we examined mis-spliced *Lr34* transcripts in adult plants, in which *Lr34r* exerts its function in resistance to pathogens. Primers Lr34-DMS-F2 and Lr34-DMS-R2 for the skipping of 92 bp at the 5’ end of exon 10, and Lr34-DMS-F3 and Lr34-DMS-R3 for the skipping of 44 bp at the 5’ end of exon 12. Four cultivars, 2174, Jagger, OK Bullet and Duster representing different genetic backgrounds, all showed the skipping of complete exon 10 (Fig 3A) and skipping of partial exon 12 (Fig 3B) of *Lr34* transcripts in flag leaves collected from plants in the field, indicating that the biological phenomenon was not unique in 2174. These cultivars showed similar proportion and patterns of mis-spliced *Lr34* transcripts, based on DNA band patterns on the gels.

## Discussion

The alternative splicing events observed in the same *Lr34r* gene from the wheat cultivar 2174 could be interpreted using common patterns described previously, including skipped or cassette exon (CE), intron retention (IR), alternative 3’ splicing sites (A3SS), as well as alternative 5’ splicing sites (A5SS) [27]. Since the RNA samples used for the *Lr34* cDNA clones may not include a mis-spliced transcript with low expression level, it is possible that many more splicing events in *Lr34* transcripts were not observed in this study. The mis-splicing events occurring in the region from exon 1 to exon 3 were observed in Thatcher isogenic lines [2], though it was not known how the transcripts were spliced out. In an analysis of ESTs deposited in GenBank, we found only two *Lr34* ESTs in the databases, both of which are from the Chinese wheat cultivar Mingxian169. One was mis-spliced due to the presence of the complete intron 6 as well as the absence of 92-bp at the 5’ end of exon 10 and 44-bp at the 5’ end of exon 12 (GenBank accession number, GU929206), and the other was mis-spliced due to the presence of the complete intron 7 as well as the absence of 44-bp at the 5’ end of exon 12 (GU929207). Obviously, the phenomenon of mis-spliced transcripts in *Lr34* in hexaploid wheat is common, but it has gone undetected in previous studies.

When a resistant *Lr34* gene was artificially mutated by EMS and the mutants that led to either alternative splice sites or mis-spliced cDNAs resulted in frame shifts, or premature stop codons, the mutant plants showed reduced resistance to leaf rust [2]. This previous study on the artificial mutant demonstrated the *Lr34* mis-splicing phenomenon at the DNA level. The present study demonstrated that even if a cultivar has a functional *Lr34* gene, the majority of its transcripts in seedlings are mis-spliced, revealing the *Lr34* mis-splicing phenomenon at the posttranscriptional level.

Almost all of the deduced proteins from the mis-spliced *Lr34* transcripts observed in this study are shorter than the truncated protein encoded by the Jagger *Lr34* allele, and they thus are unlikely to have function for resistance to leaf rust or stripe rust races previously tested. However, the possibility cannot be excluded that the proteins of various lengths deduced from the mis-spliced *Lr34* transcripts may contribute to resistance against powdery mildew or barley yellow dwarf virus. Proteins of different lengths generated from a single gene may relate functionally to resistance to diseases made by different types of pathogens [28, 29]. Functional identification of a specific Lr34 isoform by using transgenic wheat may provide clues as to whether the mis-spliced *Lr34* transcripts are involved in resistance to multiple pathogens.

The occurrence of multiple *Lr34* mis-splicing events in a single RIL made it impossible to make any association between the phenotype and a specific mis-splicing event among the RILs, because a highly susceptible reaction to leaf rust could be a consequence of different mis-splicing events. The plants treated with low temperature showed higher proportion and more forms of mis-spliced *Lr34* transcripts, compared with the control plants, indicating that the *Lr34* splicing was sensitive to low temperature (Fig 2C and 2E). Resistance of wheat seedlings to rust diseases is also sensitive to temperature. *Lr34* resistance to rust infection was increased in seedlings grown at lower temperature, as reported in previous studies [2, 4, 9]. However, it was also reported that some seedlings of Thatcher backcross lines having *Lr34* alone showed decreased resistance at lower temperature, *i*.*e*., ITs; to 3 at 5°C *vs*. ITs 2 to 3- at 20°C [30]. The inconsistency of the results on regulation of *Lr34* resistance by low temperature may be due to the genetic background of both the host lines and pathogen isolates. It is not known whether the less correctly spliced *Lr34* products could result in decreased resistance to all rust pathogens across all genetic backgrounds.

The findings in this study lead to a novel strategy of breeding cultivars resistant to rust pathogens. The mis-splicing events resulted in non-functional forms of the Lr34 protein for resistance to the rust diseases in wheat, which is a natural biological event that might be ameliorated by an understanding of mechanisms underlying mis-splicing events. Splicing of a gene is performed by an RNA and protein complex known as the spliceosome, a process regulated by a group of proteins or auxiliary elements known as exon splicing enhancers and silencers (ESEs and ESSs) and intron splicing enhancers and silencers (ISEs and ISSs) [31]. Enhancers can activate adjacent splice sites or antagonize silencers, whereas silencers can repress splice sites or enhancers. A full list of splicing regulators in humans is available, but splicing regulators in wheat are unknown and await to be identified. Further studies need to identify proteins in spliceosome that bind with *Lr34* pre-mRNA, identify exon splicing enhancers and silencers and intron splicing enhancers and silencers, identify and utilize natural mutants of negative regulators, and understand functions of various Lr34 protein isoforms in transgenic wheat.

## Conclusion

There are numerous genes/QTL that confer partial resistance to epidemic pathogens, and the innate mechanisms underlying the partial resistance are believed to be a consequence of interactions among multiple resistance genes in plants or an outcome of interactions between pathogen effectors and plant proteins. This study reported that only a portion of *Lr34* transcripts was correctly spliced in seedlings and adult plants, which could be a novel mechanism that underlies the partial resistance against leaf rust by *Lr34* in wheat. Our findings provide new insights into how the resistance of *Lr34* against rust diseases can be posttranscriptionally regulated in wheat.

## Supporting Information

S1 Fig Sequence of the complete *Lr34-B* cDNA. (DOCX)

S2 Fig Multiple alternative sites of intron 6 in *Lr34*. (DOCX)

S3 Fig Multiple sequence alignment of mis-spliced *Lr34*. (DOCX)

S1 Table Various mis-splicing events in *Lr34* transcripts. (DOCX)
