# Supplementary material for: Mis-Spliced Lr34 Transcript Events in Winter Wheat
Source: PLoS One. 2017 Jan 30;12(1):e0171149. doi: 10.1371/journal.pone.0171149 (PMC5279766; doi:10.1371/journal.pone.0171149)
Supplement: S1 Fig — (DOCX) [file pone.0171149.s001.docx]

>*Lr34-B* (LY2420)

TAGCAAAGGGCGTCGATTTAAGTCACCCATCTTGAGATGGAGGGCCTCGCGAGAGAGACCAACCCATCATCCCACCATCAAGATTTCGCCTCCTGCGCGAGTGACGAGCGCCCGGATGAGCCCGAGTTGGAATTGGCATCGCGACGGCGCCAGAATGGTGCTGGAAACAACGAGCATGTGAGTGAGAACATGCTGCTTGACAGCAGCAAGTTTGGAGCTCTCAAGAGGCGTGAGTTCTTCAACAACCTGCTAAAGAACCTCGAAGACGACCACCCCCGCTTTCTGCGCAGACAAAAGGAAAGAATTGACAGGGTTGATGTCAAGTTGCCGGCAATAGAGGTGAGGTATAATAATCTGTTTGTGGAAGCAGAGTGCAGAGTTACTAAAGGAAATCACCTGCCGTCTCTATGGAATAGTACCAAAGGTGCCTTCTCGGGCCTCGTGAAGTTGCTAGGCTTCGAAACGGAAAGAGCAAAAACCAACGTTCTAGAAGATGTCAGTGGAATCATCAAACCCTGCAGATTGACTCTTCTACTGGGACCTCCTGGATGTGGCAAAAGCACTCTGTTGCGAGCTCTTGCCGGGAAACTAGATAAATCTCTAAAGGTAACAGGGGATATCTCTTATAATTGTTATGAACTTCATGAATTTGTACCTGAGAAAACAGCTGTGTATATCAACCAACATGATCTGCACATAGCTGAGATGACTGTGAGGGAAACTTTAGACTTCTCAGCCCAGTGCCAAGGTGTTGGAAGAAGACCAAAAATACTCAAGGAGGTGAACACAAGGGAGAGTGTGGCTGGGATCATACCTGATGCGGACATCGATCTATACATGAAGGTAGTAGCAGTTGAAGCTTCAGAGCGAAGCCTACAGACAGATTATATTTTGAAGATCATGGGGCTAGAGACATGCGCAGACACGATGGTTGGGGATGCAATGAGAAGAGGAATATCAGGGGGGCAGAAGAAAAGATTAACCACAGCCGAGATGATTGTGGGACCCGCAAAAGCATACTTTATGGATGAAATATCAAATGGTCTGGATAGCTCTACCACTTTTCAAATAATCAATTGTTTCCAGCAACTGACAAACATCAGCGAGTACACGATGGTTATTTCACTTCTTCAACCAACACCTGAGGTATTTGATCTTTTCGATGACCTCATACTAATGGCAGAAGGGAAGATTATCTACCATGGCCCTCGAAATGAAGCCCTCAATCTTTTTGAGGAGTGTGGGTTCAAATGCCCAGAAAGAAAAGCGGCAGCTGACTTTCTCCAAGAGATCTTGTCCAGGAAGGACCAAGAACAGTACTGGTTGGGTCCACATGAATCATACAGATATATCTCACCTCATGAATTATCAAGCATGTTCAAGGAGAATCACAGGGGGAGAAAACTACATGAACAAAGTGTACCTCCCAAAAGCCAGTTCGGCAAGGAAGCTTTAGTATTCAATAAGTATTCGCTACGAAAACTGGAAATGTTCAAAGCCTGTGGAGCAAGGGAAGCACTCTCTAATGAAAAGGAATATGTTTGTTTATGTCTTCAAAACAGGCCAGCTTGCCATTATTGCACTCGTAACAATGTCTGTATTCCTTCGAACTCGCATGACAATAAGTTTCACTCATGCAAATTACTATATGGGAGCATTATTTTTTCCCATCTTCATGATTATGCTAAATGGCAACCAGAGATGAGCATGCAGATTGGGAGACTCCCAAGTTTTTACAAACAAAAGAGCTACTATTTCTATTCATCATGGGCATATGCAATACCAGCTTCAGTCCTAAAGGTCCCTGTTTCCATACTGGATTCGCTTGTATGGATATCTATCACATATTATGGTATTGGTTATACACATACTGTTTCAAGGTTCTTCTGCCAGTTTCTGATACTTTGTCTTCTCCATCATTCAGTCACCTCGCAGTATCGATTTATTGCTTCATACTTCCAAACACCTATTGTGTCTTTCTTCTACCTTTTTCTTGCTCTAACAGTATTCCTTACATTCGGAGGCTTCATTCTTCCCAAGACCTCCATGCCAGAATGGCTAAACTGGGGATTTTGGATATCTCCAATGGCATATGCAGAAATCAGCATAGTTATTAACGAGTTCTTGGCACCAAGATGGCAGAAGGAAAGTATTCAAAACATAACAATTGGGAACCAAATCCTGGTTAATCACGGCCTATATTACAGTTGGCATTTTTATTGGATATCCTTTGGAGCCTTGCTTGGATCTATTCTTTTATTTTATATCGCTTTTGGATTGGCACTAGATTACAGAACACCTACAGAAGAATATCATGGAAGCAGGCCTACAAAGAGCTTATGTCAACAGCAGGAAAAAGATTCCACTATTCAAAATGAATCTGATGATCAATCAAATATTTCCAAAGCAAAGATGACTATACCAACTATGCATCTTCCAATTACATTCCACAATCTGAGCTACTACATTGATACCCCACCGGAAATGCTGAAACAAGGCTATCCAACAAGAAGACTTCGACTGCTTAATAACATAACTGGAGCTTTACGTCCCGGTGTTCTTTCTGCACTAATGGGTGTTAGTGGAGCTGGGAAGACAACTCTACTAGATGTATTAGCAGGAAGGAAAACAGGAGGTTATATTGAAGGGGACATAAGAATAGGTGGATATCCCAAGGTGCAGGAAACATTTGTCAGAATCTTGGGTTACTGCGAACAAGTCGACATACATTCCCCACAGCTTACAGTTGAAGAGTCTGTAACTTATTCTGCGTGGCTTCGTCTGCCTTCTCATGTCGACAAACAAACAAGATCTAAATTTGTTGCTGAAGTCCTTGAAACTGTTGAACTAGATCAAATAAAAGATGTCTTAGTGGGGTCACCACAGAAAAATGGATTGTCCATGGAGCAGAGAAAGAGGCTAACGATTGCAGTCGAGCTTGTTTCAAACCCATCAATCATACTAATGGATGAACCAACAACAGGTTTAGATACAAGGTCAGCAGCCATTGTTATTCGTGCAGTCAAAAATATTTGTGAAACAGGAAGGACGGTAGTCTGTACAATCCATCAGCCGAGCACTGAAATTTTTGAGGCATTTGATGAGCTCATATTAATGAAAACCGGTGGGAAAACAATCTACAATGGACCAATAGGAGAGCGCTCCTGCAAAGTGATTGAGTACTTTGAGAAAATTTCTGGAGTCCCAAAAATAAAGAGTAACTGCAATCCAGCTACCTGGATGATGGATGTAACATCGACATCAATGGAGGTTCAACACAACATGGACTTTGCAATTTTGTATGAAGAGTCGTCACTGCATAGAGAAGCTGAAGATCTAGTGGAGCAGCTAAGTATCCCATTACCAAATTCAGAAAATCTACGCTTCTCCCATAGTTTTGCACAGAATGGCTGGATTCAACTTAAAGCTTGCTTGTGGAAACAAAACATAACTTACTGGAGAAGTCCTCAGTATAACTTGAGGCGCATTATGATGACTGTCATATCTGCCCTGATCTACGGAGTATTGTTCTGGAAGCATGCAAAAGTATTAAACAACGAGCAGGACATGCTCAGTGTTTTTGGTGCAATGTATTTGGGTTTCACAACCATAGGCGCTTATAATGATCAGACAATCATACCATTCAGTACGACTGAGCGTATTGTAATGTATCGTGAGAAATTTGCAGGAATGTATTCATCTTGGTCATATTCATTCGCACAGGCTTTCATTGAGATACCCTATGTATTTATCCAAGTGGTACTGTATACGTTAATTGTCTATCCGTCAACTGGTTATTATTGGACAGCACACAAATTCCTATGGTTCTTCTACACTACATTTTGTTCAATTCTCTCCTATGTTTATGTTGGGTTGCTTCTTGTTTCAATAACCCCCAATGTTCAAGTAGCTACCATACTGGCTTCATTTTTCAACACCATGCAAACACTATTCTCAGGATTTATTTTACCTGCACCTCAAATCCCAAAGTGGTGGACTTGGCTCTACTATCTCACTCCTACATCTTGGGCACTCAATGCCCTCTTGACATCACAATACGGAAACATAGAAAAAGAGGTGAAAGCATTTGGAGAAACTAAATCAGTTTCAATCTTCTTGAATGACTATTTTGGGTTTCATCAAGACAAGTTGAGCATAGTAGCAACTGTCCTCGTTGCCTTTCCTTTTGTGTTGATAATCTTGTTTTCGTTGTCCATTGAGAAACTTAATTTCCAGAAGAGGTAAGCAAGTTCTGACATTCCAACAG

**S1 Fig. Lr34-B cDNA sequence.** Primers Lr34-Exp-F1 (5’-TAGCAAAGGGCGTCGATTTA-3’) and Lr34-Exp-R1 (5’- CTGTTGGAATGTCAGAACTTGC-3’) were used to amplify cDNA of *Lr34* genes from wheat cultivar 2174. Twenty of 43 clones sequenced using vector primers M13 showed cDNAs from *Lr34-B* on chromosome 4A. One of the 20 clones was completely sequenced (clone: LY2420), and its sequence is provided above (Fig. S1). Primer sequences are highlighted in green, and the start codon and stop codon for translation are highlighted in yellow.
