# Supplementary material for: Mis-Spliced Lr34 Transcript Events in Winter Wheat
Source: PLoS One. 2017 Jan 30;12(1):e0171149. doi: 10.1371/journal.pone.0171149 (PMC5279766; doi:10.1371/journal.pone.0171149)
Supplement: S2 Fig — (DOCX) [file pone.0171149.s002.docx]

**b**

**c**


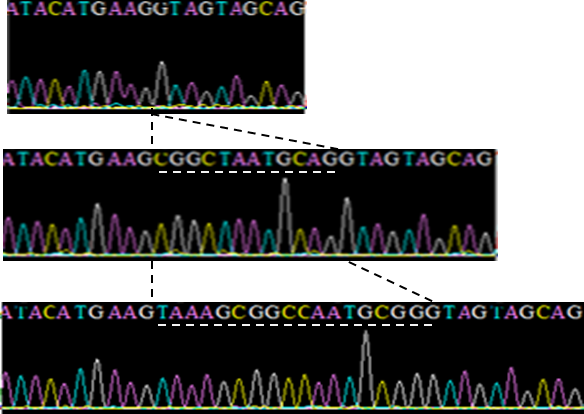


**a**

**S2 Fig. Alternative sites of intron 6 in** ***Lr34***. **(A)**, Intron 6 was correctly spliced out and exon 6 and exon 7 were joined. **(B)**, The underlined 12 bp at the 3’ end of intron 6 were retained (YL105 and LY4289). **(C)**, The underlined 17 bp at the 3’ end of intron 6 were retained (LY4235).
