# Supplementary material for: Mis-Spliced Lr34 Transcript Events in Winter Wheat
Source: PLoS One. 2017 Jan 30;12(1):e0171149. doi: 10.1371/journal.pone.0171149 (PMC5279766; doi:10.1371/journal.pone.0171149)
Supplement: S3 Fig — (DOCX) [file pone.0171149.s003.docx]

**S3 Fig. A multiple sequence alignment of Lr34 cDNA sequences.** Twenty-three cDNA clones containing *Lr34* on chromosome 7D were completely sequenced at Oklahoma State University. The sequences were aligned using the Bio-Edit program (Fig. S3). Lr34CDNA indicates a clone that has correctly spliced *Lr34* from 2174, and other sequence numbers indicate the numbers of clones that were completely sequenced. Dot indicates identical sequence, dash indicates locations of skipped exons or retained introns. SNPs were caused by Taq polymerase during PCRs or sequencing errors.
