# Supplementary material for: Mis-Spliced Lr34 Transcript Events in Winter Wheat
Source: PLoS One. 2017 Jan 30;12(1):e0171149. doi: 10.1371/journal.pone.0171149 (PMC5279766; doi:10.1371/journal.pone.0171149)
Supplement: S1 Table — (DOCX) [file pone.0171149.s004.docx]

**S1 Table. Various mis-splicing events in *Lr34* transcripts**

| **Clone code #** | **Mis-splicing type** | **Mis-splicing site** | **Size (bp)** |
| --- | --- | --- | --- |
| LY2879  LY2637  LY2638  LY2874  LY2881  LY3195  LY3343  LY3347  LY3353  LY3912  LY4228  LY4235  LY4289  LY4817  YL105 | Intron retention  Exon skipping  Intron retention  Exon skipping  Exon skipping  Exon skipping  Intron retention  Exon skipping  Exon skipping  Exon skipping  Exon skipping  Exon skipping  Exon skipping  Exon skipping  Intron retention  Intron retention  Exon skipping  Intron retention | Complete intron 1  Partial exon 10 at 5’ end  Complete intron 9 retention  Partial exon 12 at 5’ end  Partial exon 10 at 5’ end  Partial exon 12 at 5’ end  Complete Intron 14  Complete exon16  Partial exon 12 at 5’ end  Partial exon 12 at 5’ end  Partial exon 10 at 5’ end  Partial exon 12 at 5’ end  Partial exon 12 at 5’ end  Partial exon 10 at 5’ end  Complete exon 12  Partial intron 6 at 3’ end  Partial intron 6 at 3’ end  Partial exon 10 at 5’ end  Partial intron 6 at 3’ end | 99  92  92  44  92  44  437  74  44  44  92  44  44  92  161  17  12  11  12 |

|  |  |  |  |
| --- | --- | --- | --- |
|  |  |  |  |
